# Supplementary material for: Modelling the impact of interventions on the progress of the COVID-19 outbreak including age segregation
Source: PLoS One. 2021 Mar 15;16(3):e0248243. doi: 10.1371/journal.pone.0248243 (PMC7959345; doi:10.1371/journal.pone.0248243)
Supplement: S1 File — (PDF) [file pone.0248243.s001.pdf]

S1 File

Modelling the impact of interventions on the progress of the COVID-19 outbreak including age segregation

Jorge Rodríguez[1*], Mauricio Patón[1], Joao M Uratani[1] and Juan M Acuña[2]

[1]Department of Chemical Engineering. College of Engineering. Khalifa University.
SAN Campus PO Box 2533 Abu Dhabi, United Arab Emirates

[2]Department of Epidemiology and Public Health. College of Medicine. Khalifa University.
PO Box 127788 Abu Dhabi, United Arab Emirates

[*]Corresponding author: *jorge.rodriguez@ku.ac.ae*

Section I. Epidemiological and clinical parameters per age group

Table S1. Default epidemiological and clinical parameters per age group used in the COVID-19 outbreak case study simulations presented.

Parameter	0s	10s	20s	30s	40s	50s	60s	70s	80+
f_{ps_ni}	1	1	1	1	1	1	1	1	1
f_{s_ps}	0.692	0.692	0.692	0.692	0.692	0.692	0.692	0.692	0.692
f_{sh_s}	0.0054	0.0033	0.0144	0.0310	0.0443	0.0755	0.1213	0.1840	0.3600
f_{sc_sh}	0.140	0.083	0.060	0.072	0.085	0.111	0.147	0.259	0.480
f_{d_sc}	0.051	0.217	0.258	0.230	0.272	0.391	0.669	0.861	0.989
f_{r_ni}	$1- f_{ps\_ni}$	$1- f_{ps\_ni}$	$1- f_{ps\_ni}$	$1- f_{ps\_ni}$	$1- f_{ps\_ni}$	$1- f_{ps\_ni}$	$1- f_{ps\_ni}$	$1- f_{ps\_ni}$	$1- f_{ps\_ni}$
f_{r_ps}	$1- f_{s\_ps}$	$1- f_{s\_ps}$	$1- f_{s\_ps}$	$1- f_{s\_ps}$	$1- f_{s\_ps}$	$1- f_{s\_ps}$	$1- f_{s\_ps}$	$1- f_{s\_ps}$	$1- f_{s\_ps}$
f_{r_s}	$1- f_{sh\_s}$	$1- f_{sh\_s}$	$1- f_{sh\_s}$	$1- f_{sh\_s}$	$1- f_{sh\_s}$	$1- f_{sh\_s}$	$1- f_{sh\_s}$	$1- f_{sh\_s}$	$1- f_{sh\_s}$
f_{r_sh}	$1- f_{sc\_sh}$	$1- f_{sc\_sh}$	$1- f_{sc\_sh}$	$1- f_{sc\_sh}$	$1- f_{sc\_sh}$	$1- f_{sc\_sh}$	$1- f_{sc\_sh}$	$1- f_{sc\_sh}$	$1- f_{sc\_sh}$
f_{r_sc}	$1- f_{d\_sc}$	$1- f_{d\_sc}$	$1- f_{d\_sc}$	$1- f_{d\_sc}$	$1- f_{d\_sc}$	$1- f_{d\_sc}$	$1- f_{d\_sc}$	$1- f_{d\_sc}$	$1- f_{d\_sc}$
t_{ps_ni}	4	4	4	4	4	4	4	4	4
t_{s_ps}	1.5	1.5	1.5	1.5	1.5	1.5	1.5	1.5	1.5
t_{sh_s}	7	7	7	7	7	7	7	7	7
t_{sc_sh}	5	5	5	5	5	5	5	5	5
t_{d_sc}	11	11	11	11	11	11	11	11	11
t_{d_nc}	1	1	1	1	1	1	1	1	1
t_{r_ni}	1	1	1	1	1	1	1	1	1
t_{r_ps}	3	3	3	3	3	3	3	3	3
t_{r_s}	9	9	9	9	9	9	9	9	9
t_{r_sh}	14	14	14	14	14	14	14	14	14
t_{r_sc}	10	10	10	10	10	10	10	10	10

Section II. Data sources for the epidemiological and clinical parameters: COVID-19 case study

Table S2. Data sources and level of confidence assigned to the epidemiological and clinical parameters from Table S1 for the COVID-19 outbreak case study.

Parameter	Sources and details of estimation	Confidence Level
f_{ps_ni}	Estimated (100%)	L
f_{s_ps}	Reported [1,2]	L
f_{sh_s}	Reported [2,3]	VL
f_{sc_sh}	Reported [3]	M
f_{d_sc}	Reported [3]	M
f_{r_ni}	Estimated (0%)	M
f_{r_ps}	Calculated ($1 - f_{s\_ps}$)	M
f_{r_s}	Calculated ($1 - f_{sh\_s}$)	M
f_{r_sh}	Calculated ($1 - f_{sc\_sh}$)	M
f_{r_sc}	Calculated ($1 - fd_{\_sc}$)	M
t_{ps_ni}	Estimated by personal communication	L
t_{s_ps}	Reported [4]	L
t_{sh_s}	Reported [5,6]	M
t_{sc_sh}	Reported [6]	M
t_{d_sc}	Personal communications	M
t_{d_nc}	Estimated as one day	M
t_{r_ni}	Estimated by analogy	VL
t_{r_ps}	Estimated by analogy	VL
t_{r_s}	Estimated by analogy	L
t_{r_sh}	Reported [6]	M
t_{r_sc}	Personal communications	M

References

1. Nishiura H, Kobayashi T, Miyama T, Suzuki A, Jung S, Hayashi K, et al. Estimation of the asymptomatic ratio of novel coronavirus infections (COVID-19). International Journal of Infectious Diseases. 2020;94: 154–155. doi:10.1016/j.ijid.2020.03.020
2. Pollán M, Pérez-Gómez B, Pastor-Barriuso R, Oteo J, Hernán MA, Pérez-Olmeda M, et al. Prevalence of SARS-CoV-2 in Spain (ENE-COVID): a nationwide, population-based seroepidemiological study. The Lancet. 2020;396: 535–544. doi:10.1016/S0140-6736(20)31483-5
3. ISCIII. Situación de COVID-19 o Coronavirus en España. 30 Apr 2020 [cited 10 May 2020]. Available: https://covid19.isciii.es/
4. Lauer SA, Grantz KH, Bi Q, Jones FK, Zheng Q, Meredith HR, et al. The Incubation Period of Coronavirus Disease 2019 (COVID-19) From Publicly Reported Confirmed Cases: Estimation and Application. Annals of Internal Medicine. 2020 [cited 29 Apr 2020]. doi:10.7326/M20-0504
5. Bendix A. A day-by-day breakdown of coronavirus symptoms shows how COVID-19 goes from bad to worse. In: Business Insider [Internet]. [cited 4 Nov 2020]. Available: https://www.businessinsider.com/coronavirus-covid19-day-by-day-symptoms-patients-2020-2
6. Zhou F, Yu T, Du R, Fan G, Liu Y, Liu Z, et al. Clinical course and risk factors for mortality of adult inpatients with COVID-19 in Wuhan, China: a retrospective cohort study. The Lancet. 2020;395: 1054–1062. doi:10.1016/S0140-6736(20)30566-3

Section III. Behavioural and intervention parameters per age group: COVID-19 case study

Table S3. Behavioural and intervention parameter values per age as selected for the case study

Parameter*	0s	10s	20s	30s	40s	50s	60s	70s	80+
$\mathbf{ni_h}$	10	10	10	10	10	10	10	10	10
$\mathbf{lpa_h}$	0.1	0.25	0.5	0.5	0.5	0.5	0.5	0.5	0.5
$\mathbf{lpa_{ps}}$	0.1	0.25	0.5	0.5	0.5	0.5	0.5	0.5	0.5
$\mathbf{lpa_s}$	0.1	0.5	0.75	0.75	0.75	0.75	0.75	0.75	0.75
$\mathbf{rfi_s}$	0.5	0.5	0.5	0.5	0.5	0.5	0.5	0.5	0.5

*Rationale: The default level of personal protection and awareness (**lpa**) in children and youngsters is taken as smaller than that of adults; Adult symptomatic individuals are expected to take higher level of personal protection and awareness ($\mathbf{lpa_s}$) to not spread any general disease to others irrespective of the knowledge of their specific condition. No reduction factor of their social interactivity respect to healthy ones is applied for pre-symptomatic infected individuals as they are ignorant of their condition; Symptomatic infected individuals are expected to reduce their social interactivity respect to healthy ones as they feel sick ($\mathbf{rfi_s} < 1$); a sensitivity of the tests for PS is set at 80% and for S at 85%.

Section IV. COVID-19 outbreak simulation under no interventions

The model simulation of the outbreak time course under the default parameters and no intervention is presented in Figure S4.

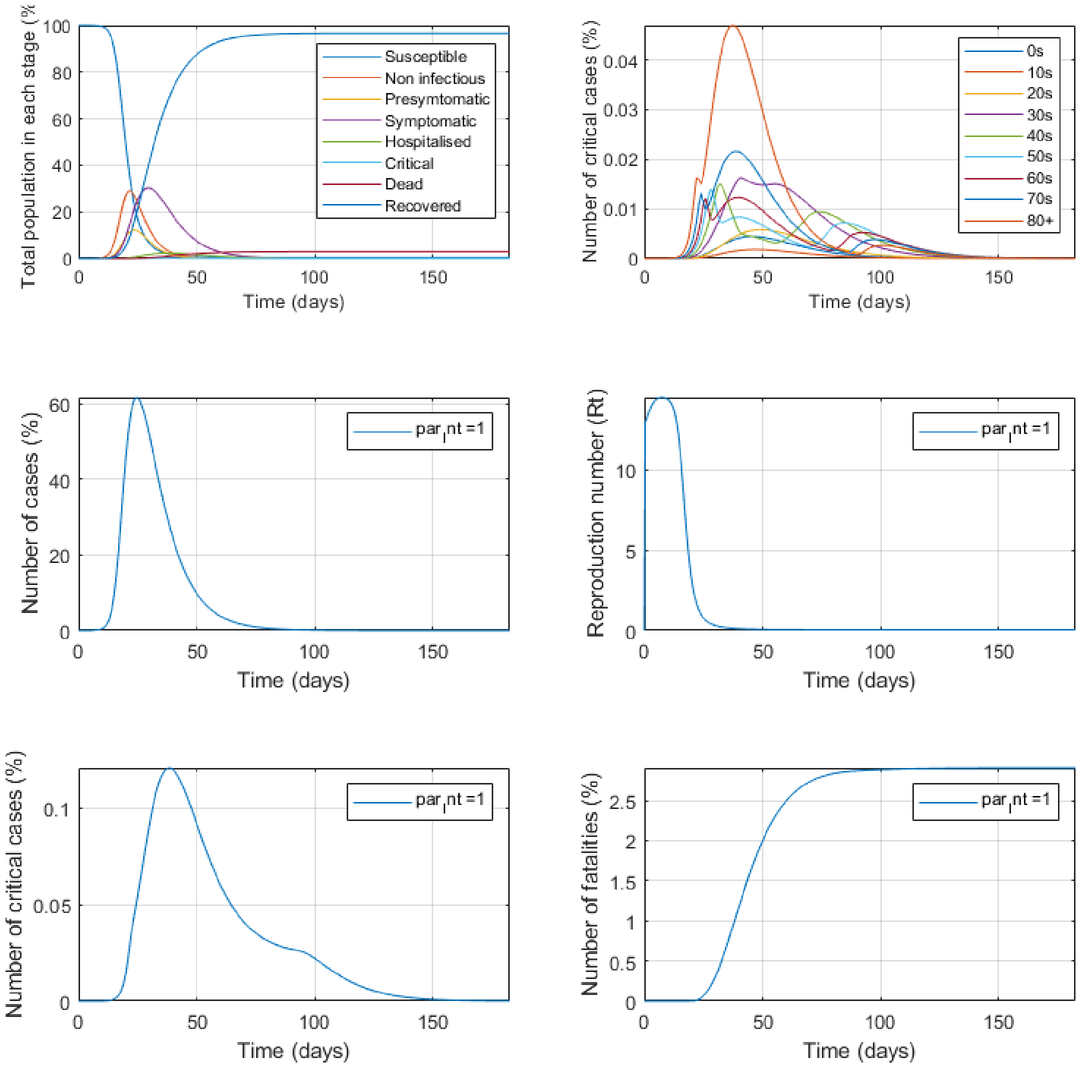

Figure S4. Model simulation of the COVID-19 outbreak under no interventions using the default parameters in S1 and S3. The time course profiles of population in each stage is presented (top) as well as those for the total number active cases (middle left); reproduction number (R_t) (middle right); the number of critical cases (bottom left) and the number of fatalities (bottom right). Numbers are in percentage of the total population of all ages.

Section V. Matrix of state transitions

The matrix represents the transitions between states of the disease and compartments.

Stages \ Rates	ri_ps (#infPS/d)	ri_s (#infS/d)	rps_ni (#NI-PS/d)	rs_ps (#PS-S/d)	rsh_s (#S-SH/d)	rsc_sh (#SH-SC/d)	rd_sc (#SC-D/d)	rr_ni (#NI-R/d)	rr_ps (#PS-R/d)	rr_s (#S-R/d)	rr_sh (#SH-R/d)	rr_sc (#SC-R/d)
N_h	-1	-1	0	0	0	0	0	0	0	0	0	0
N_{ni}	1	1	-1	0	0	0	0	-1	0	0	0	0
N_{ps}	0	0	1	-1	0	0	0	0	-1	0	0	0
N_s	0	0	0	1	-1	0	0	0	0	-1	0	0
N_{sh}	0	0	0	0	1	-1	0	0	0	0	-1	0
N_{sc}	0	0	0	0	0	1	-1	0	0	0	0	-1
N_d	0	0	0	0	0	0	1	0	0	0	0	0
N_r	0	0	0	0	0	0	0	1	1	1	1	1

Figure S5a. Matrix of state transitions as governed by the infection and transition rates defined.

Section VI. Dynamic simulation results under social isolation interventions

Impact of universal social isolation

The dynamic simulation results of the impact of social isolation of all population are shown in Figure S6.a.

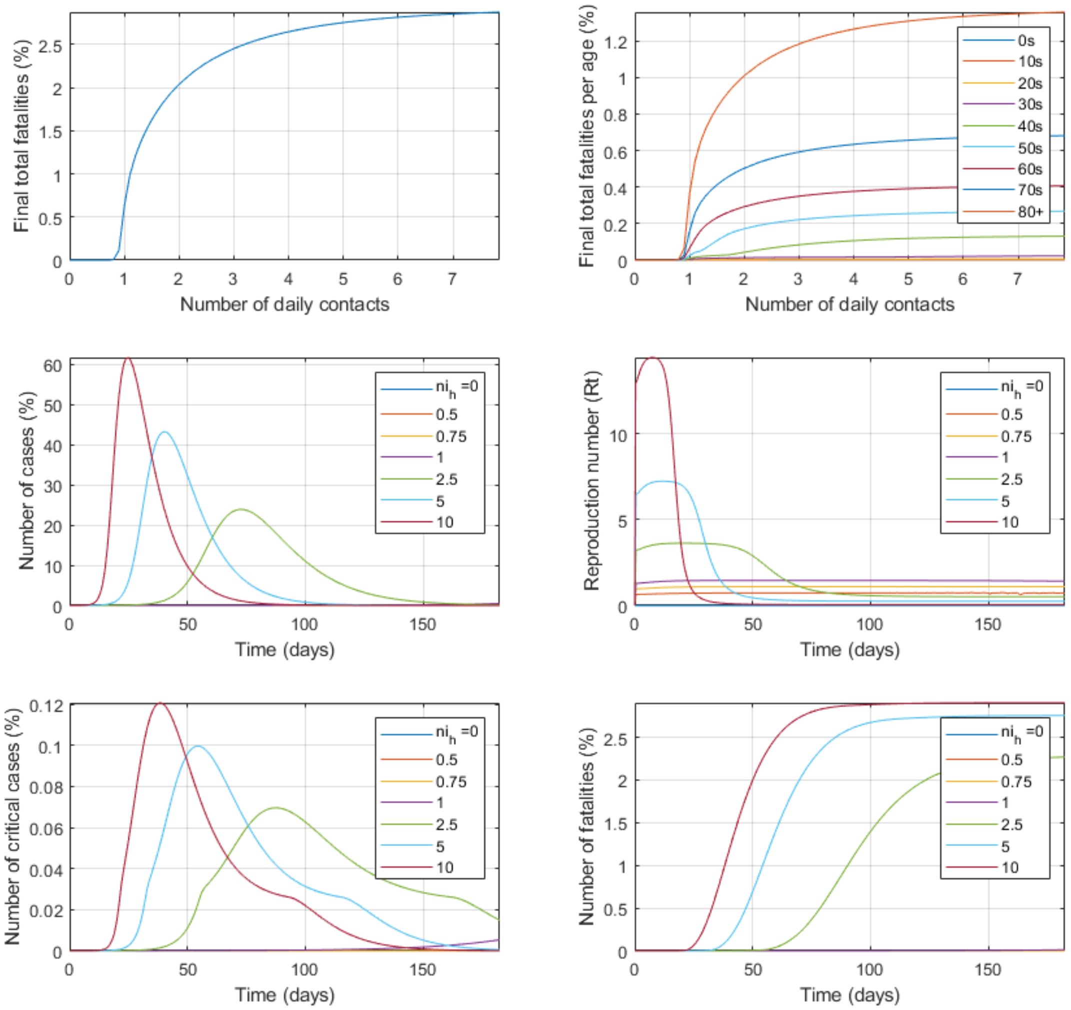

Figure S6.a. Impact of universal social isolation on the final total number of fatalities (top left); the final total number of fatalities per age group (top right) as well as for the different time course profiles of the total active cases (middle left); reproduction number (R_t) (middle right); the number of critical cases (bottom left) and the number of fatalities (bottom right). Numbers are as percentage of total population of all ages.

Impact of social isolation of the elderly only

The dynamic simulation results of the impact of social isolation of only the elderly individuals are shown in Figure S6.b.

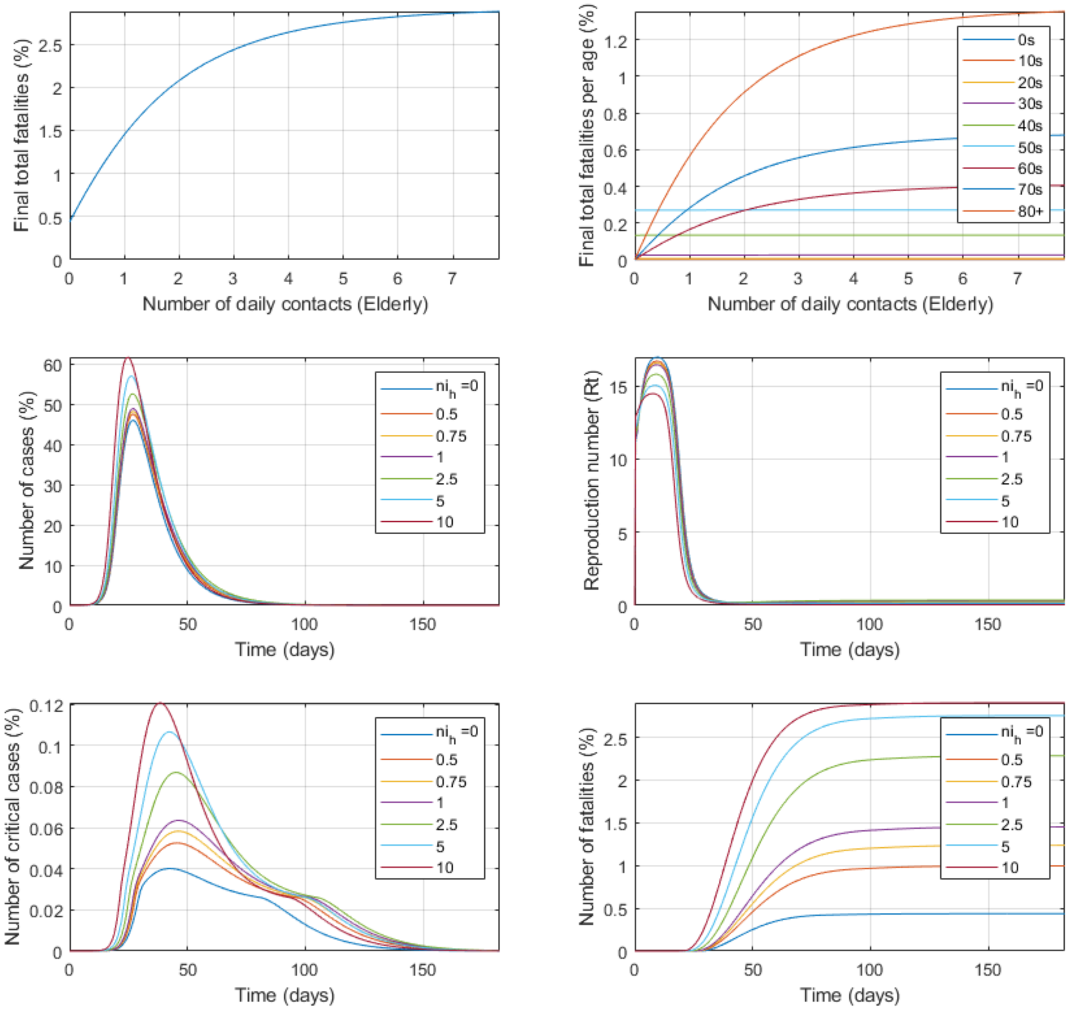

Figure S6.b. Impact of selective social isolation measures for the elderly only on the final total number of fatalities (top left); the final total number of fatalities per age group (top right) as well as for the different time course profiles of the total active cases (middle left); reproduction number (R_t) (middle right); the number of critical cases (bottom left) and the number of fatalities (bottom right). Numbers are in percentage of the total population of all ages.

Impact of social isolation of the young only

The dynamic simulation results of the impact of social isolation of only the young individuals are shown in Figure S6.c.

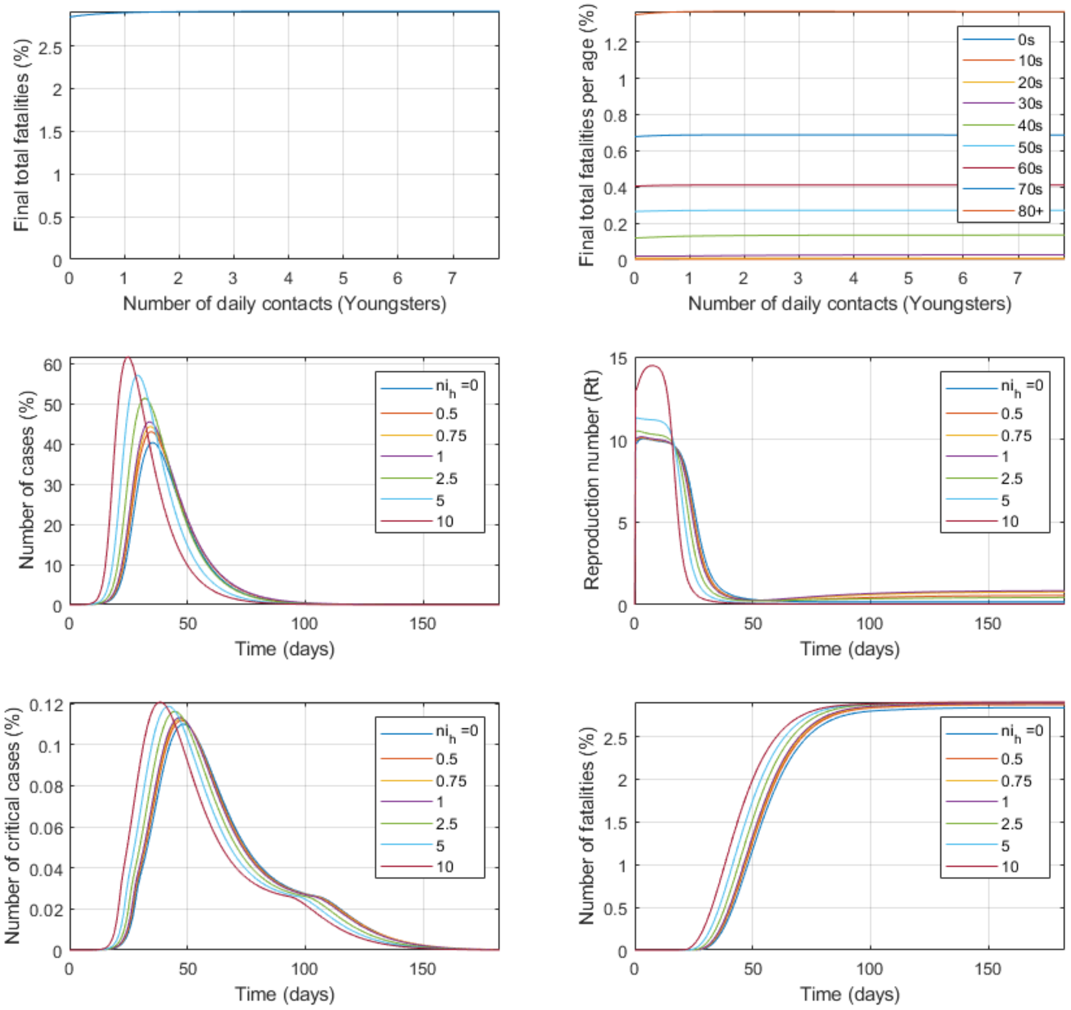

Figure S6.c. Impact of selective social isolation to the young on the final total number of fatalities (top left); the final number of fatalities per age group (top right) as well as for the different time course profiles of total active cases (middle left); reproduction number (R_t) (middle right); number of critical cases (bottom left) and the number of fatalities (bottom right). Numbers are in percentage of the total population.

Impact of social isolation of the elderly and young only

The dynamic simulation results of the impact of social isolation of only the young and elderly individuals are shown in Figure S6.d.

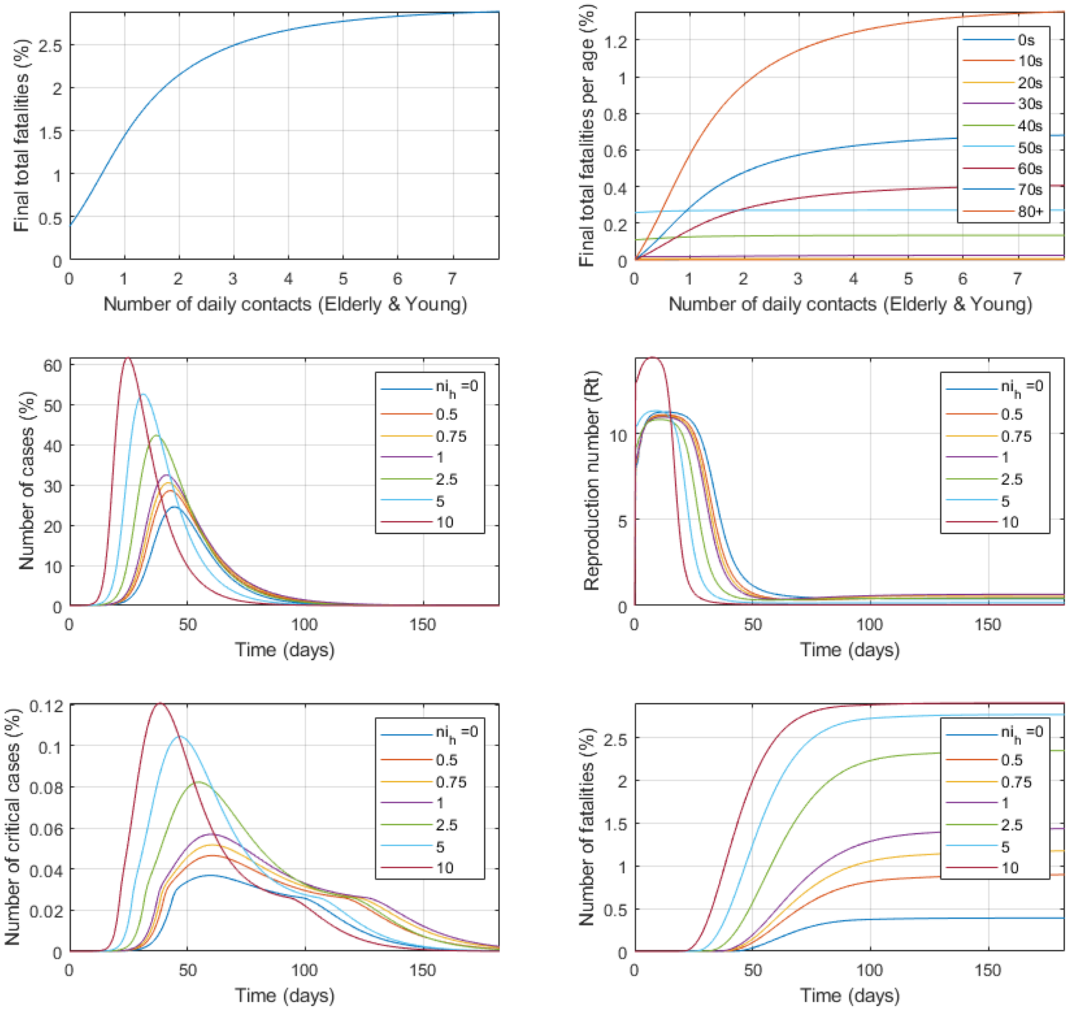

Figure S6.d. Impact of selective social isolation measures applied to both for the young and the elderly only but not to the rest of the population on the final total number of fatalities (top left); the final total number of fatalities per age group (top right) as well as for the different time course profiles of the total active cases (middle left); reproduction number (R_t) (middle right); the number of critical cases (bottom left) and the number of fatalities (bottom right). Numbers are in percentage of the total population of all ages.

Section VII. Dynamic simulation results under increased use of PPE and distancing

The dynamic simulation results of the impact of increased use of PPE and distancing are shown in Figure S7.

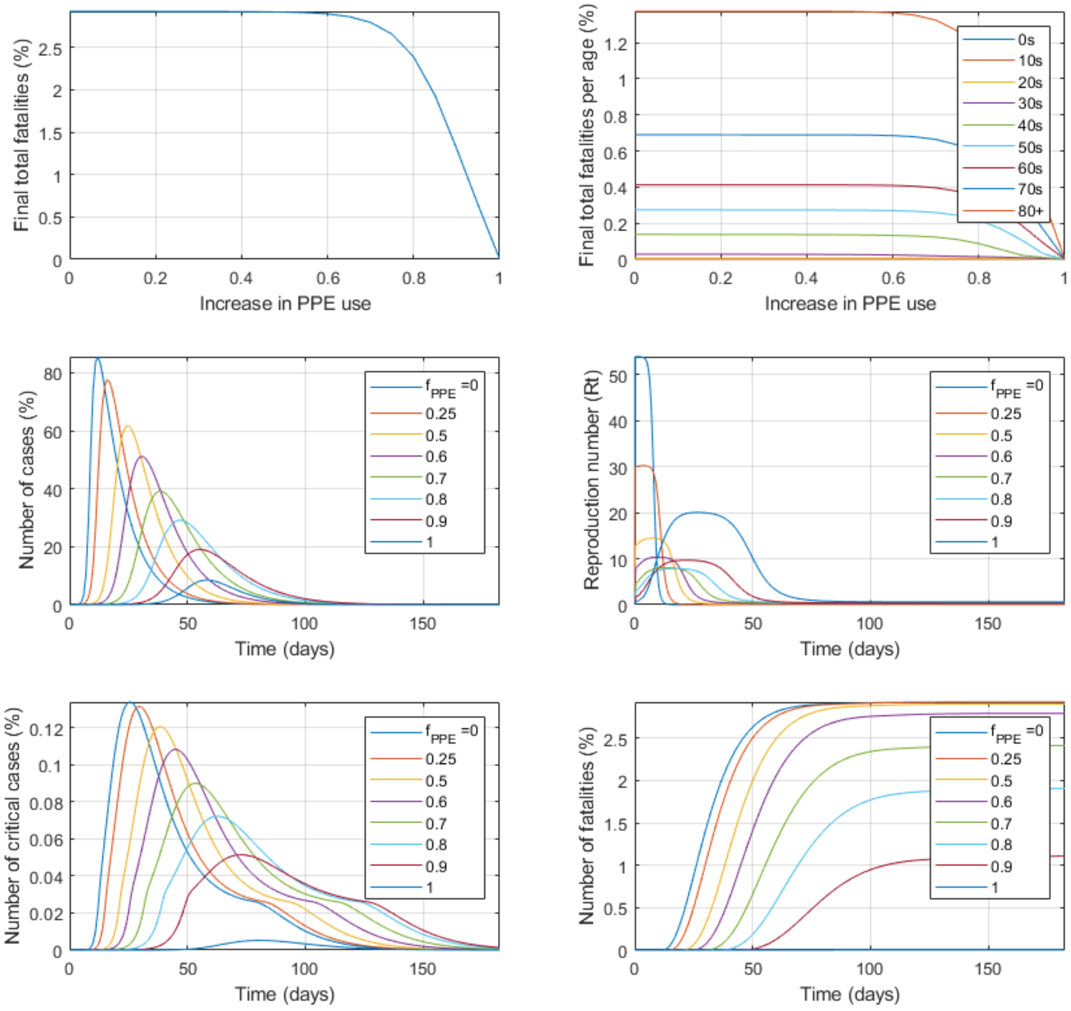

Figure S7. Impact of an increase factor in the use of PPE by both infected and healthy groups respect to the default values (Table S3) on the final total number of fatalities (top left); the final total number of fatalities per age group (top right) as well as for the different time course profiles of the total active cases (middle left); reproduction number (R_t) (middle right); the number of critical cases (bottom left) and the number of fatalities (bottom right). Numbers are in percentage of the total population of all ages.

Section VIII. Dynamic simulation results for increased awareness of infection and quarantine

Testing of symptomatic individuals

The dynamic simulation results of the impact of testing of symptomatic individuals shown in Figure S8.a.

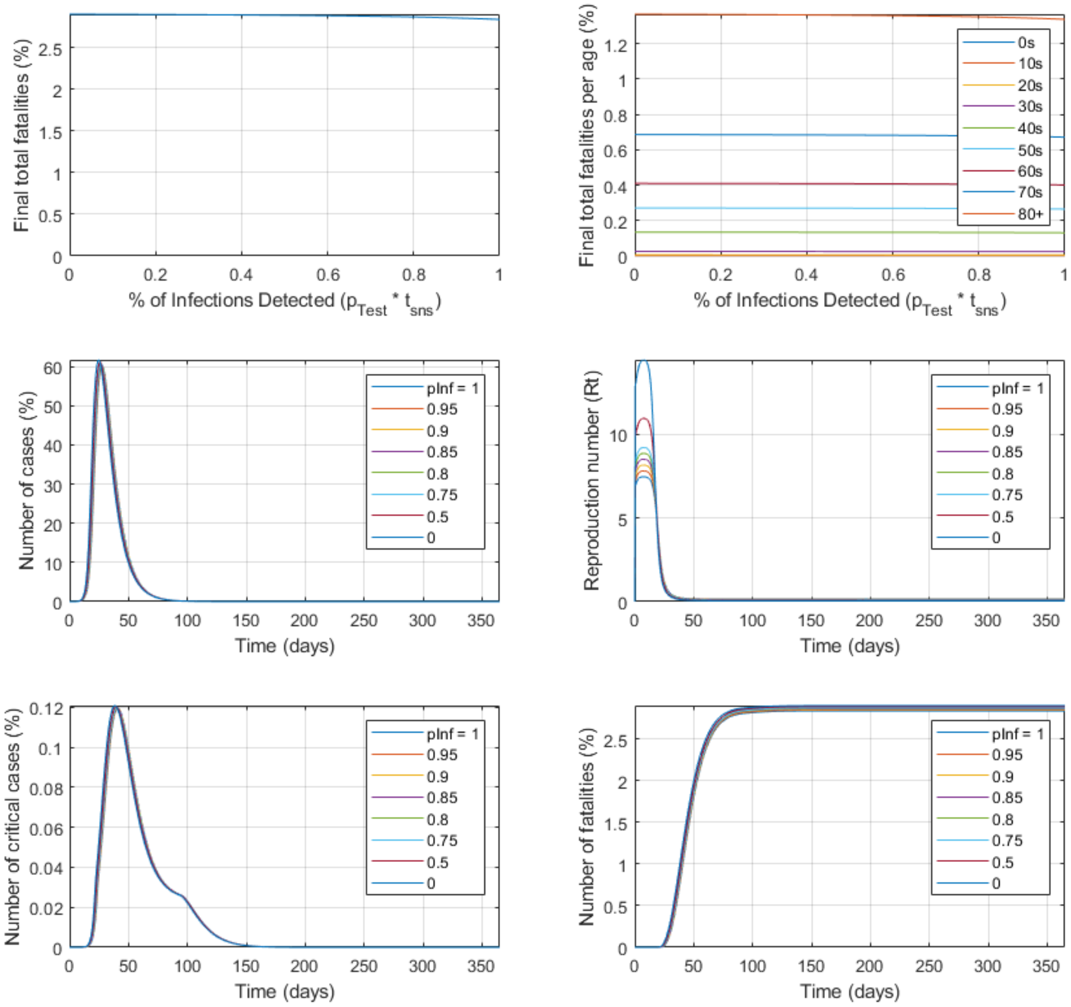

Figure S8a. Impact of extensive testing of the infected symptomatic individuals only that leads to modified isolation factor respect to their default rfi values (Table S3), on the final total number of fatalities (top left); the final total number of fatalities per age group (top right) as well as for the different time course profiles of the total active cases (middle left); reproduction number (R_t) (middle right); the number of critical cases (bottom left) and the number of fatalities (bottom right). Numbers are in percentage of the total population of all ages.

Testing of non-symptomatic individuals

The dynamic simulation results of the impact of testing of non-symptomatic individuals shown in Figure S8.b.

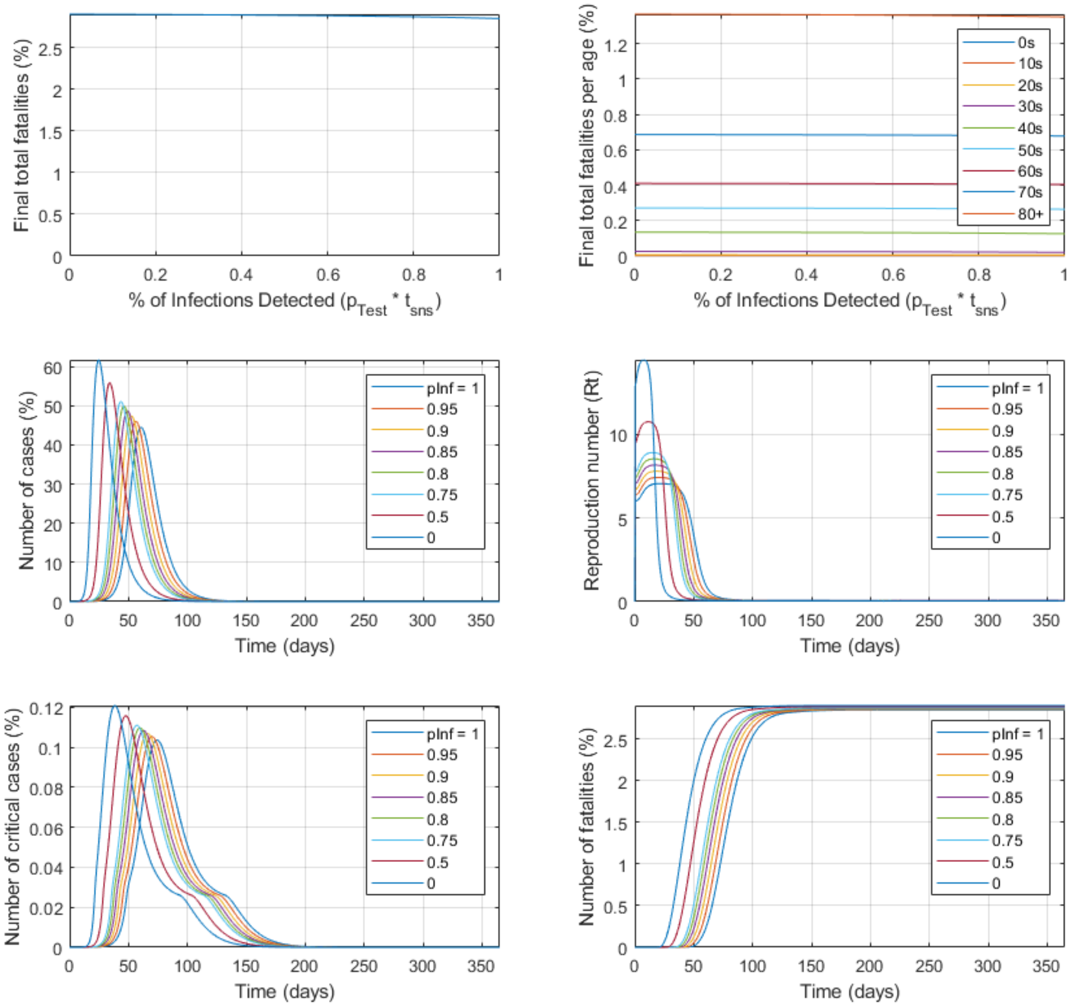

Figure S8b. Impact of extensive testing of the infected pre-symptomatic individuals only that leads to modified isolation factor respect to their default rfi values (Table S3), on the final total number of fatalities (top left); the final total number of fatalities per age group (top right) as well as for the different time course profiles of the total active cases (middle left); reproduction number (R_t) (middle right); the number of critical cases (bottom left) and the number of fatalities (bottom right). Numbers are in percentage of the total population of all ages.

Testing of all individuals

The dynamic simulation results of the impact of testing of both symptomatic and non-symptomatic individuals shown in Figure S8.c.

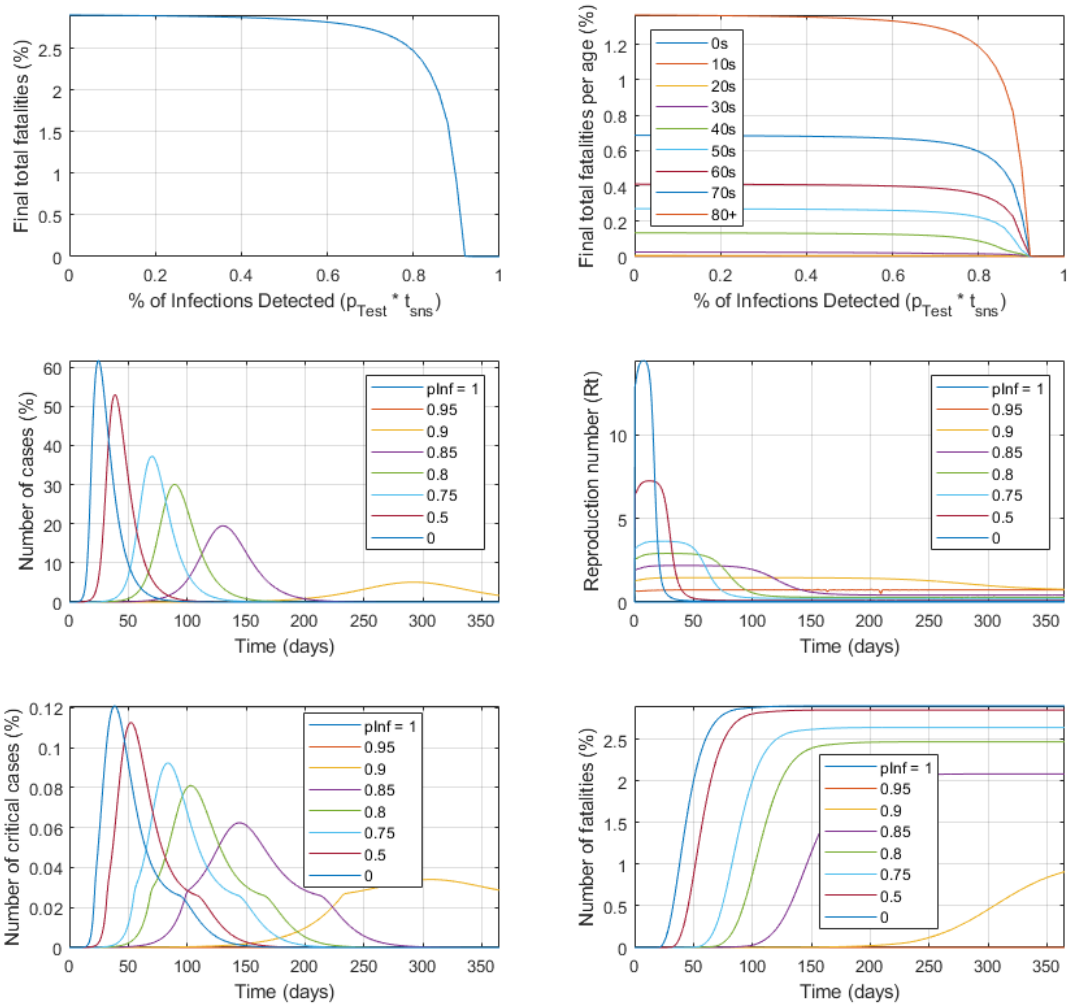

Figure S8c. Impact of extensive testing of both infected symptomatic and pre-symptomatic individuals that leads to modified isolation factor respect to their default rfi values (Table S3), on the final total number of fatalities (top left); the final total number of fatalities per age group (top right) as well as for the different time course profiles of the total active cases (middle left); reproduction number (R_t) (middle right); the number of critical cases (bottom left) and the number of fatalities (bottom right). Numbers are in percentage of the total population of all ages.

Section IX. Dynamic simulation results for increased critical health care capacity

The dynamic simulation results of the impact of increased critical health care capacity are shown in Figure S9.

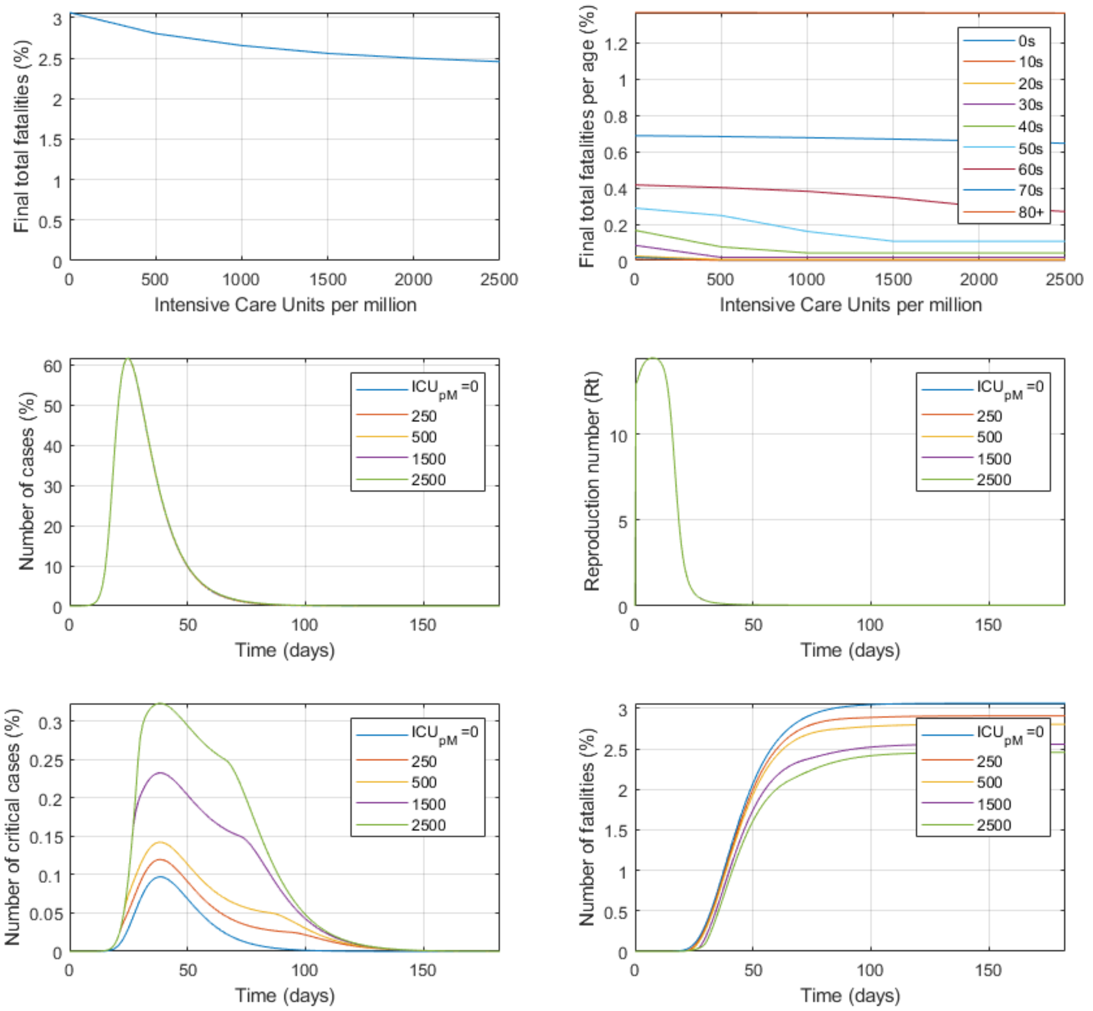

Figure S9. Impact of the availability of intensive care beds on the final total number of fatalities (top left); the final total number of fatalities per age group (top right) as well as for the different time course profiles of the total active cases (middle left); reproduction number (R_t) (middle right); the number of critical cases (bottom left) and the number of fatalities (bottom right). Numbers are in percentage of the total population of all ages.

Section X. Sensitivity analysis for the epidemiological and clinical parameters

A sensitivity analysis for the model parameters is presented in terms of the impact that changes in the values of each parameter respect to its default value has on the curves of final total predicted fatalities at the outbreak conclusion for four intervention cases, namely (i) the universal social isolation; (ii) the selective social isolation of the elderly; (iii) the increase of use of PPE and social distancing and (iv) the percetage of infections detected. Each parameter evaluated is decreased and increased by a factor multiplication as indicated.

The most sensitive transition times are tsh,s and tsc,sh. Faster times of transition increase the total number of fatalities as the sick person moves faster to more severe stages with lower recovery expected. On the other hand, the transition of non-infectious to pre-symptomatic and from critical to deceased have a negligible impact on the simulation results. Analogously to the transition fractions, the recovery times for the critical cases appear to be less impactful than from less advanced stages of the disease.

Epidemiological parameters related to severity and mortality

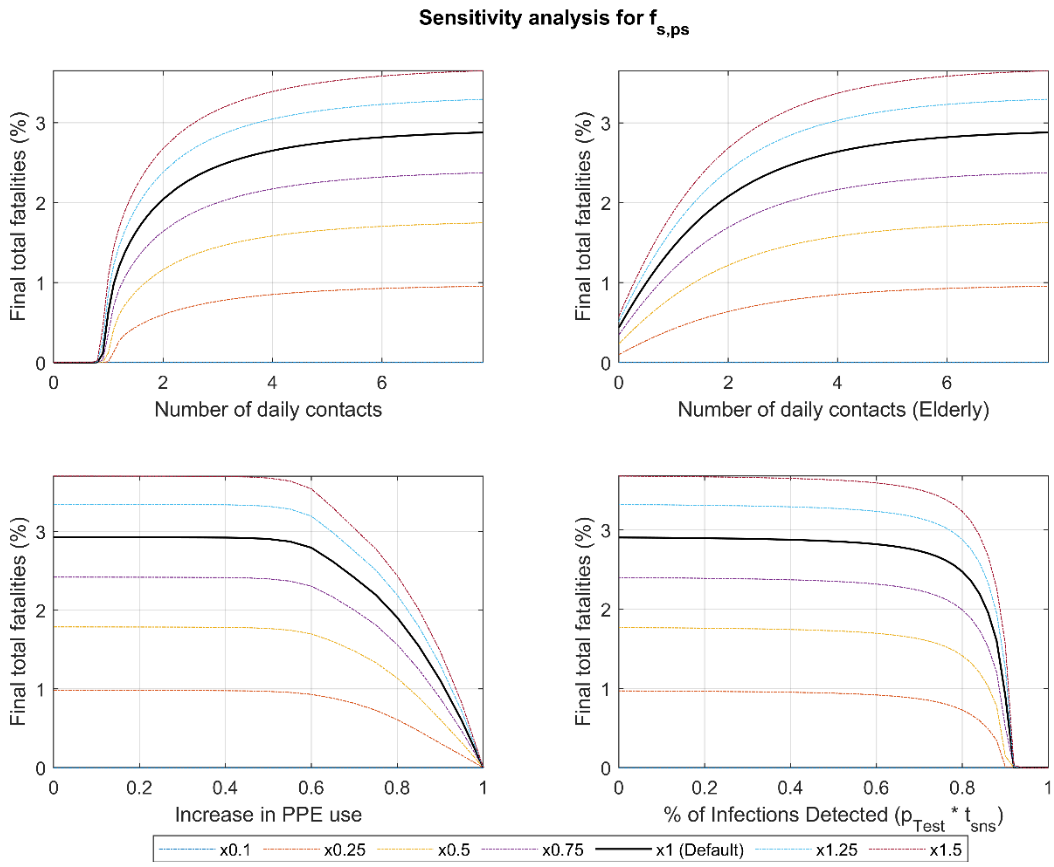

Figure S10.1. Sensitivity analysis for $f_{s,ps}$. The value used for the parameter is the one shown in Table S1 multiplied by the corresponding value shown in the legend.

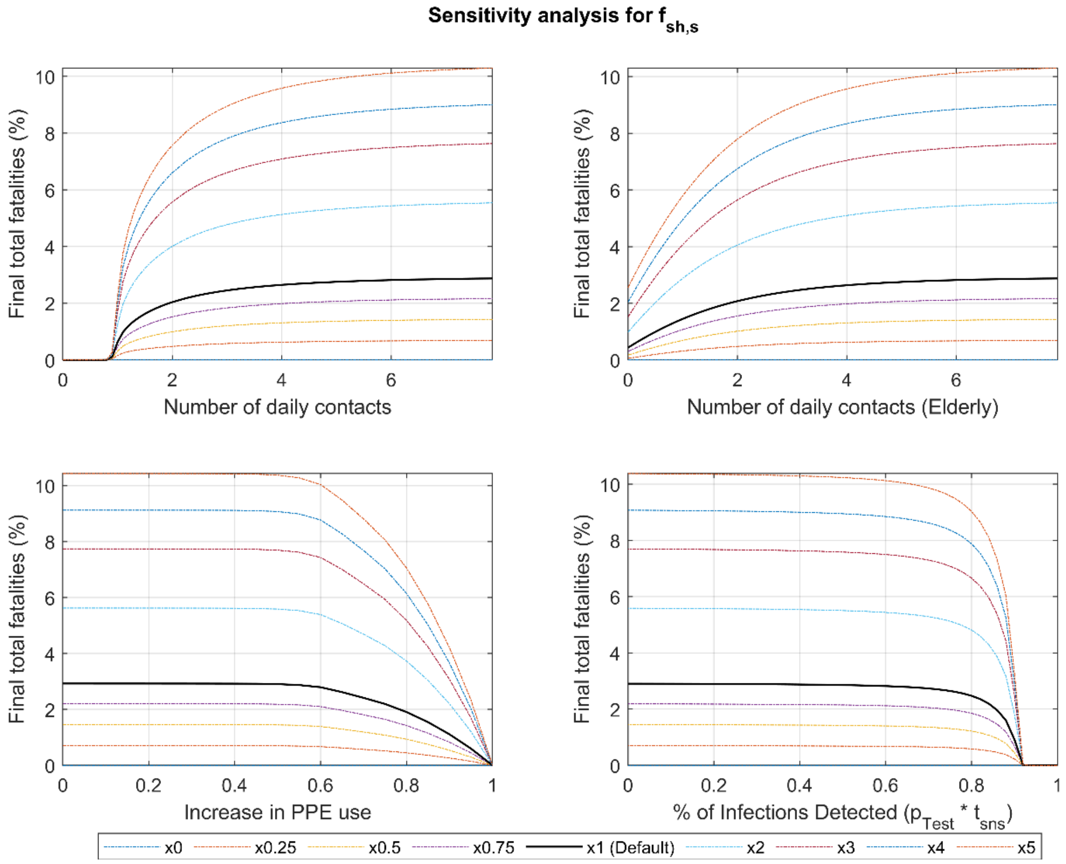

Figure S10.2. Sensitivity analysis for $f_{sh,s}$. The value used for the parameter is the one shown in Table S1 multiplied by the corresponding value shown in the legend.

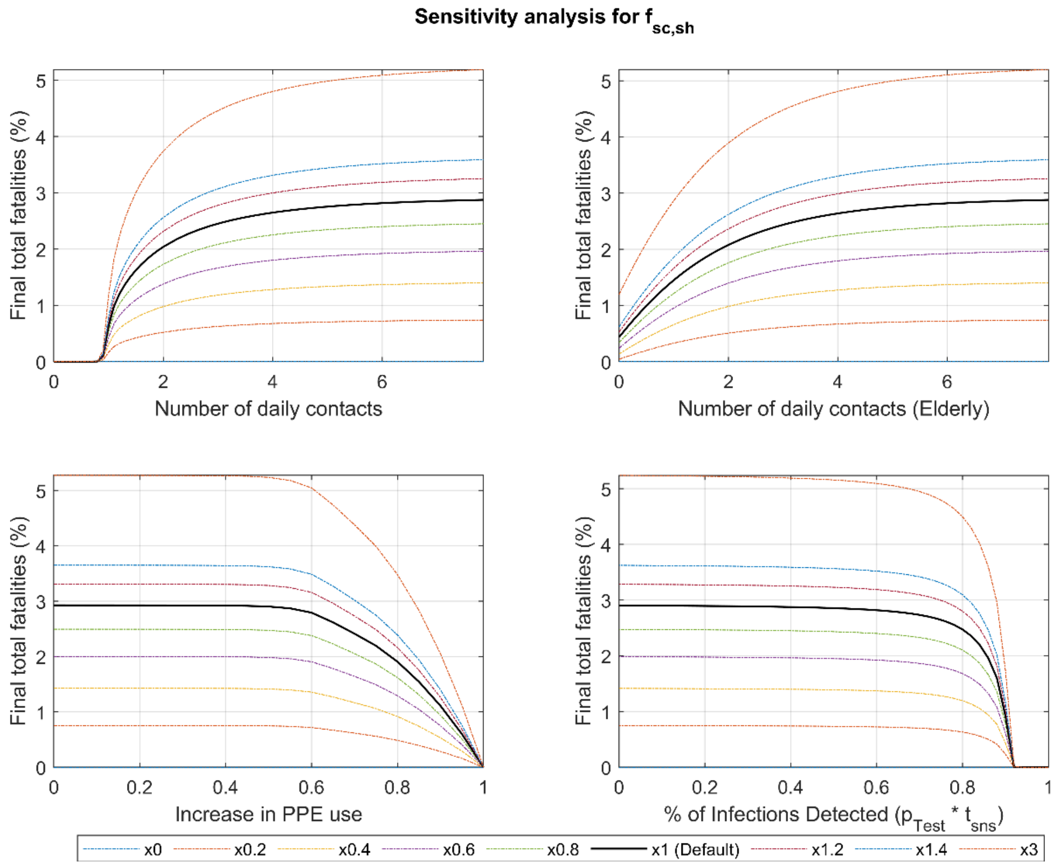

Figure S10.3. Sensitivity analysis for $f_{sc,sh}$. The value used for the parameter is the one shown in Table S1 multiplied by the corresponding value shown in the legend.

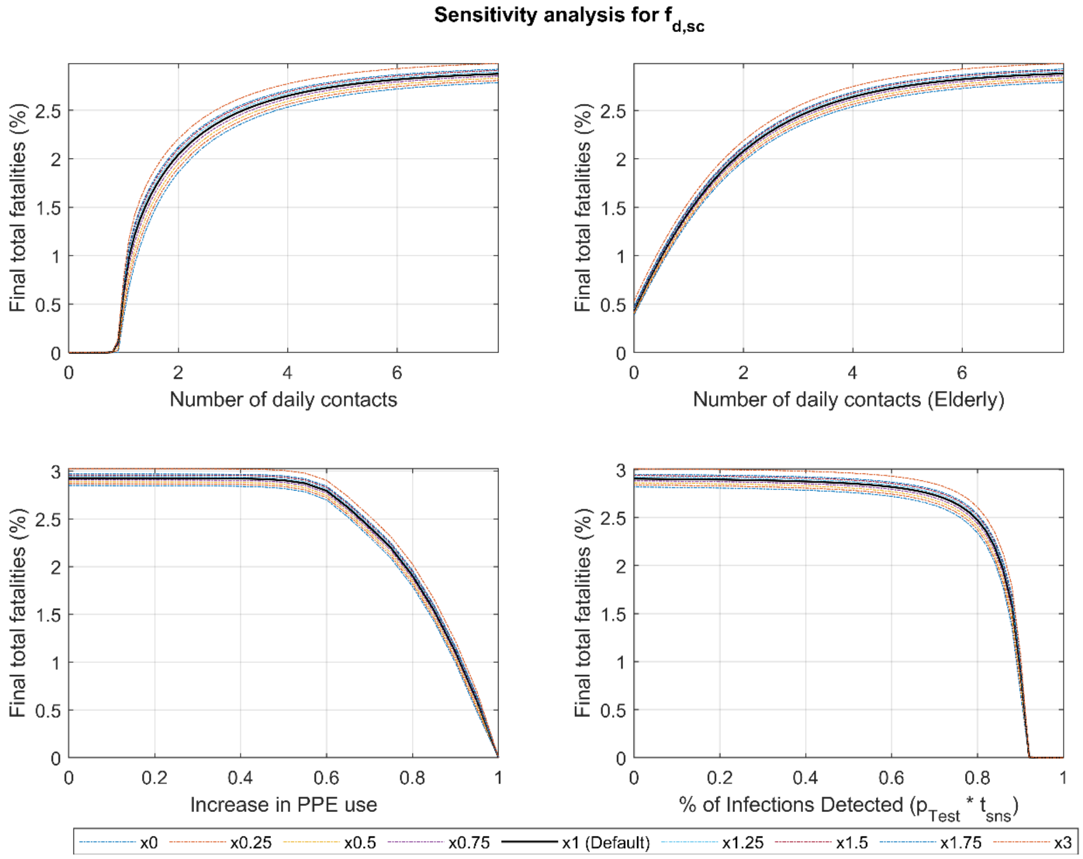

Figure S10.4. Sensitivity analysis for $f_{d,sc}$. The value used for the parameter is the one shown in Table S1 multiplied by the corresponding value shown in the legend.

Times at each state of infection and recovery

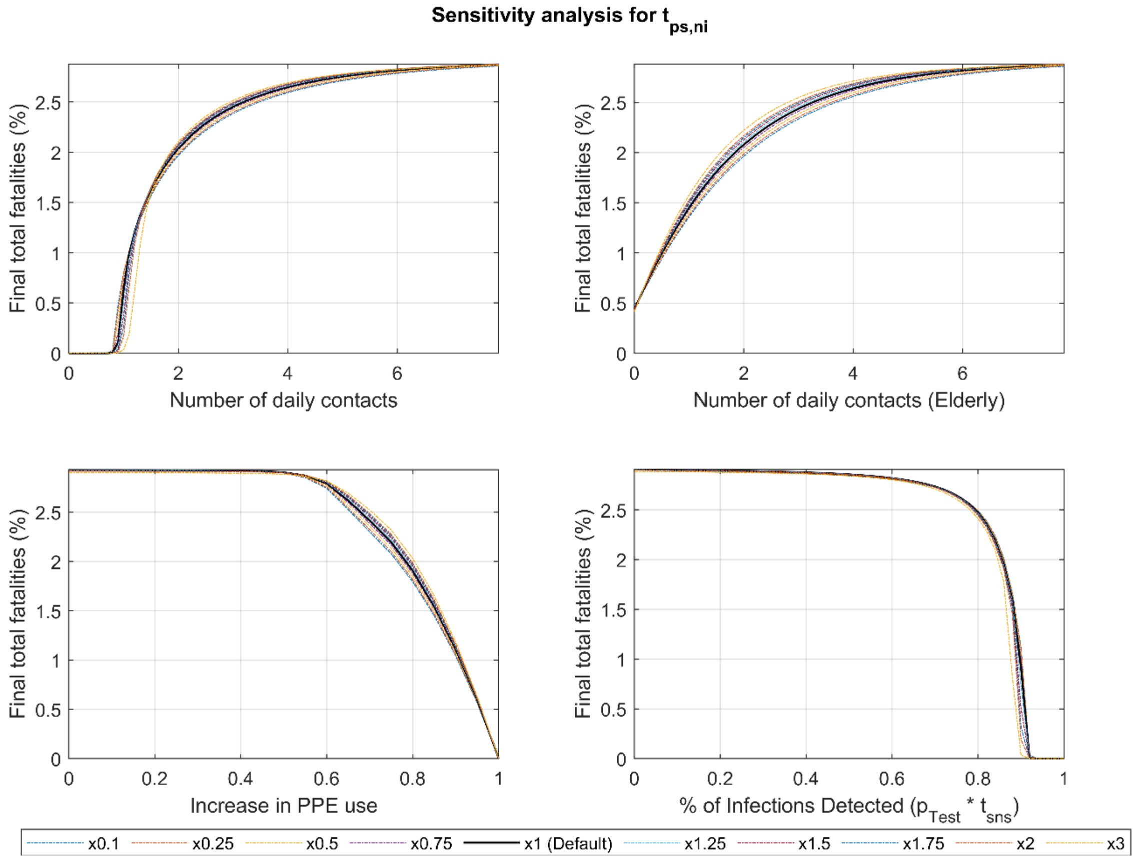

Figure S10.5. Sensitivity analysis for $t_{ps,ni}$. The value used for the parameter is the one shown in Table S1 multiplied by the corresponding value shown in the legend.

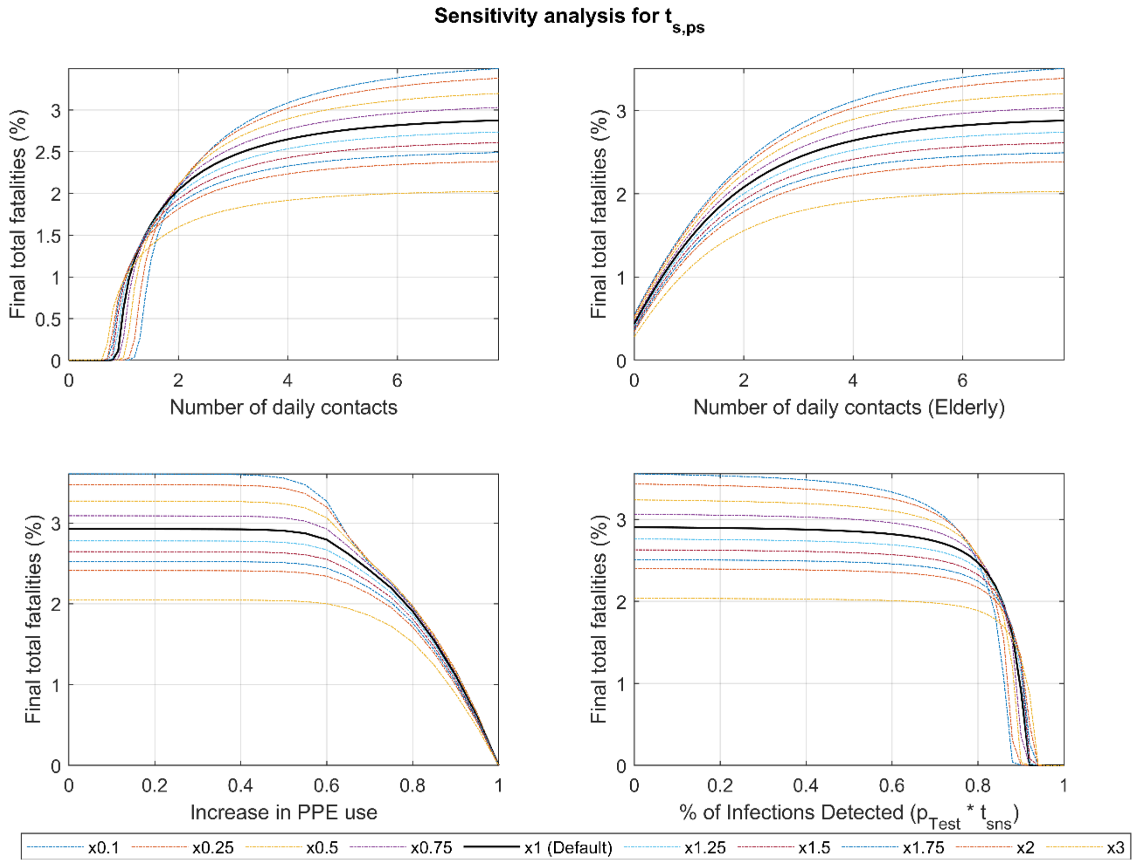

Figure S10.6. Sensitivity analysis for $t_{s,ps}$. The value used for the parameter is the one shown in Table S1 multiplied by the corresponding value shown in the legend.

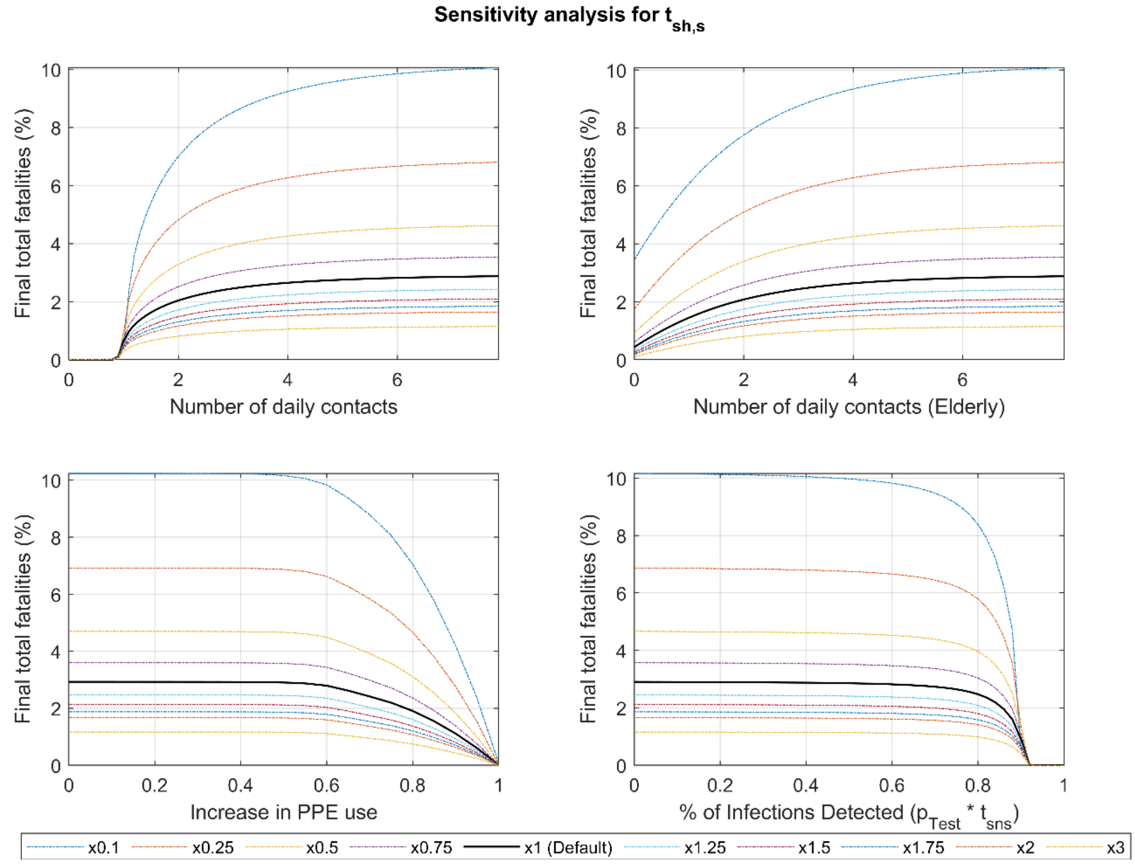

Figure S10.7. Sensitivity analysis for $t_{sh,s}$. The value used for the parameter is the one shown in Table S1 multiplied by the corresponding value shown in the legend.

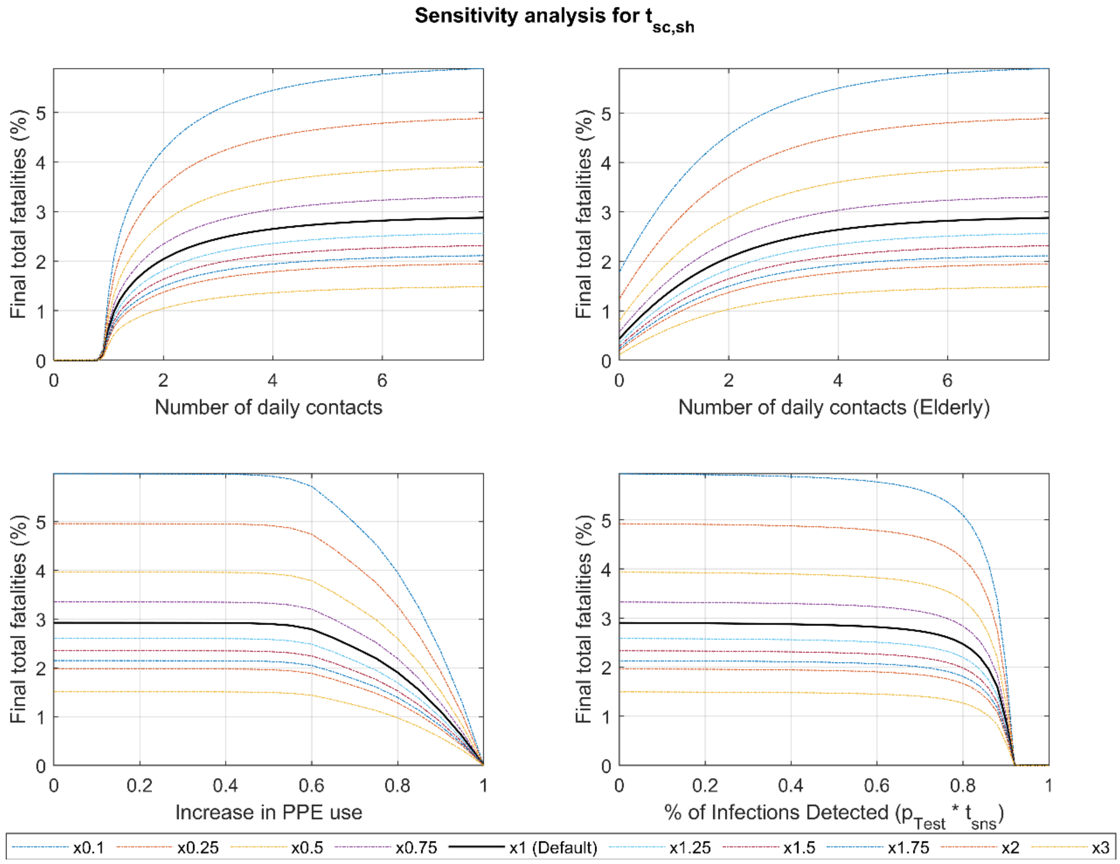

Figure S10.8. Sensitivity analysis for $t_{sc,sh}$. The value used for the parameter is the one shown in Table S1 multiplied by the corresponding value shown in the legend.

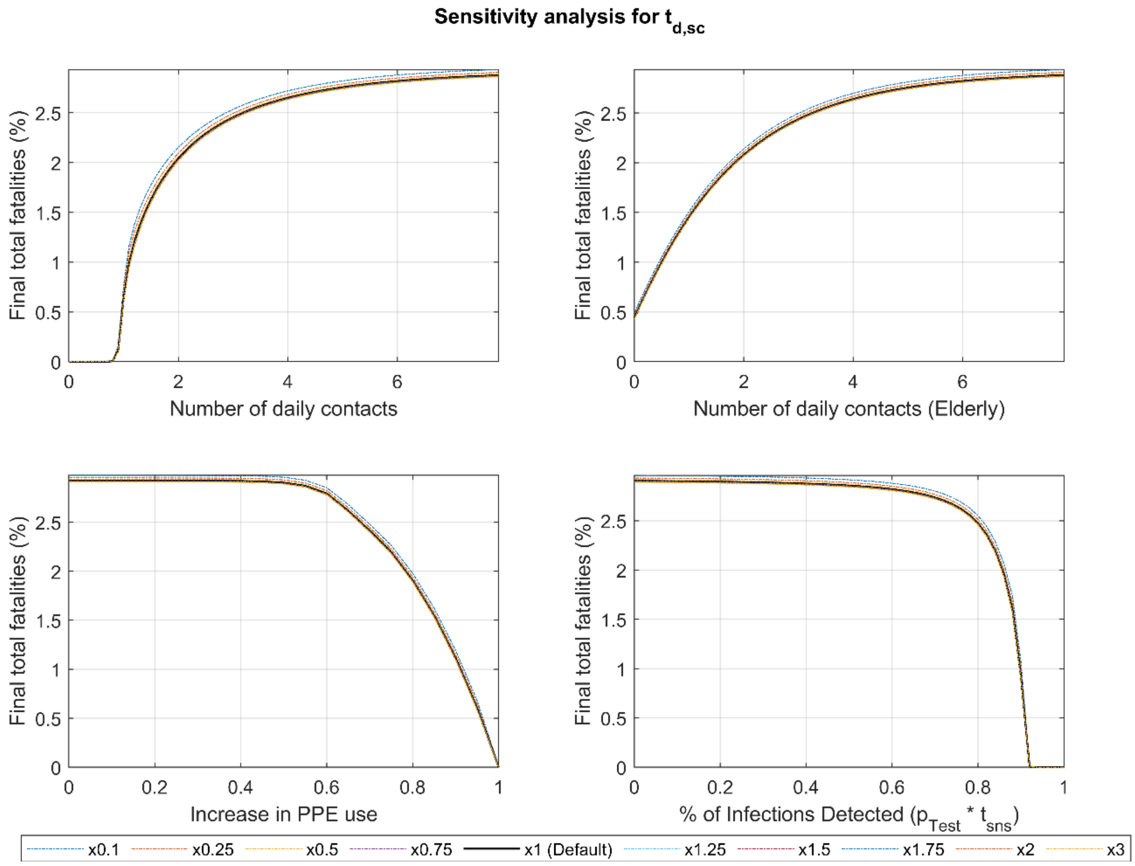

Figure S10.9. Sensitivity analysis for $t_{d,sc}$. The value used for the parameter is the one shown in Table S1 multiplied by the corresponding value shown in the legend.

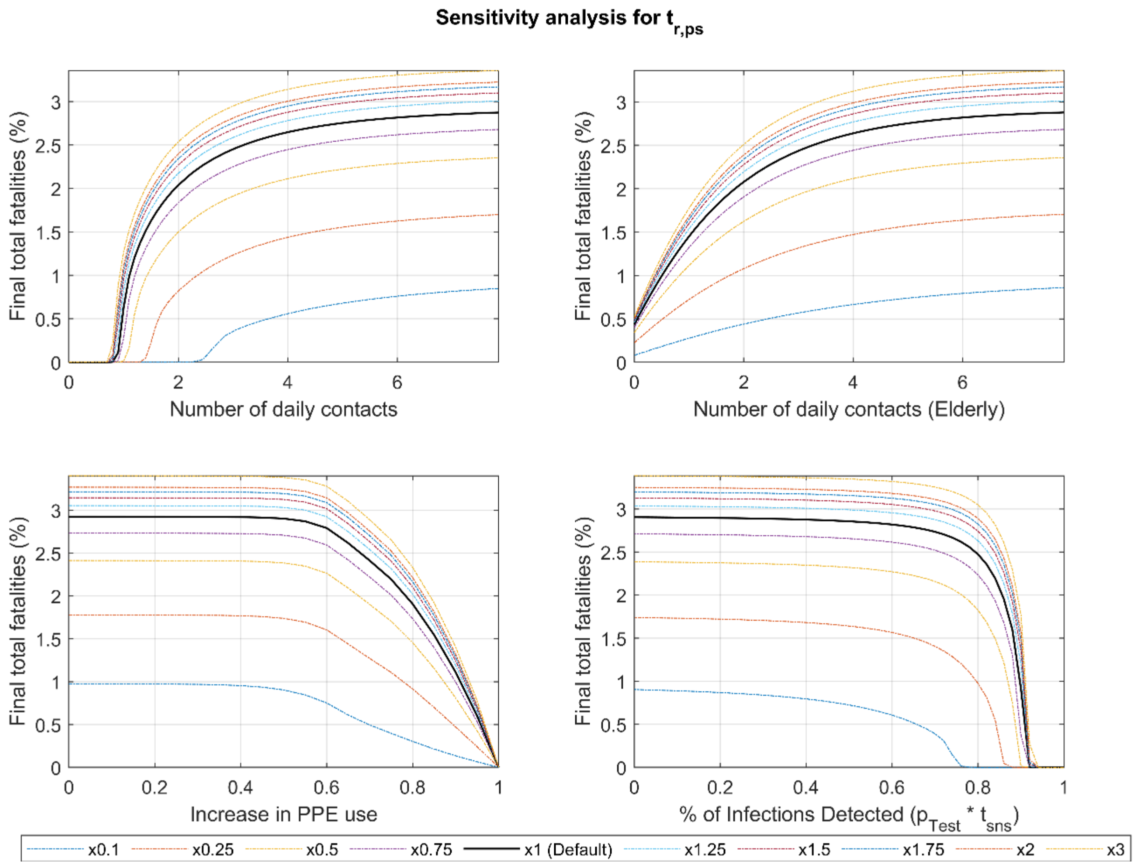

Figure S10.10. Sensitivity analysis for $t_{r,ps}$. The value used for the parameter is the one shown in Table S1 multiplied by the corresponding value shown in the legend.

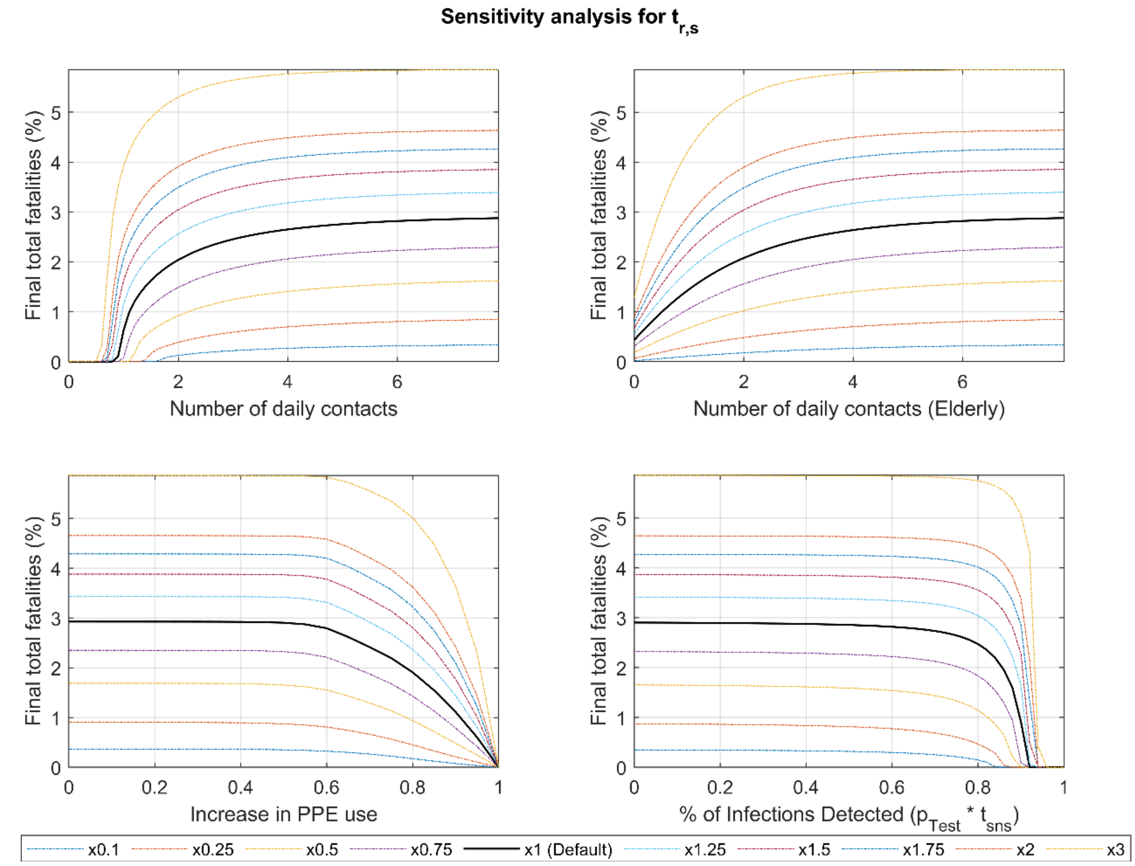

Figure S10.11. Sensitivity analysis for $t_{r,s}$. The value used for the parameter is the one shown in Table S1 multiplied by the corresponding value shown in the legend.

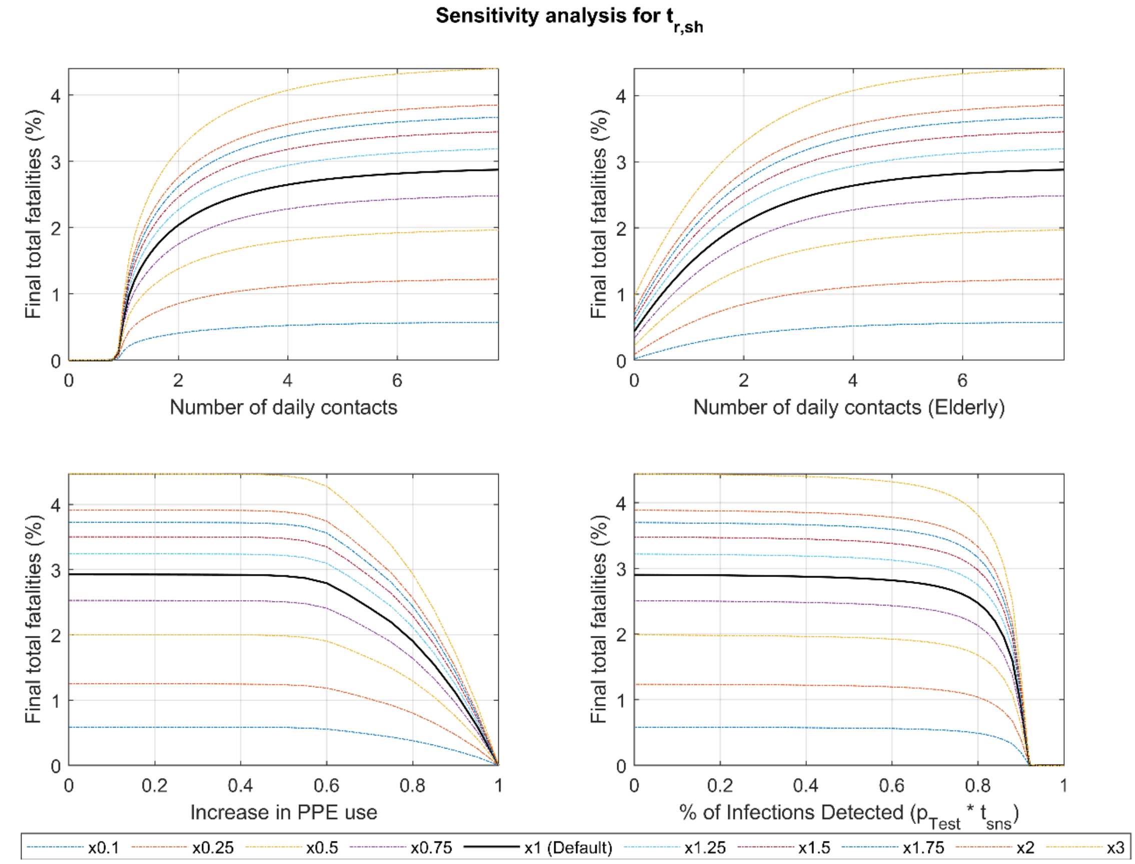

Figure S10.12. Sensitivity analysis for $t_{r,sh}$. The value used for the parameter is the one shown in Table S1 multiplied by the corresponding value shown in the legend.

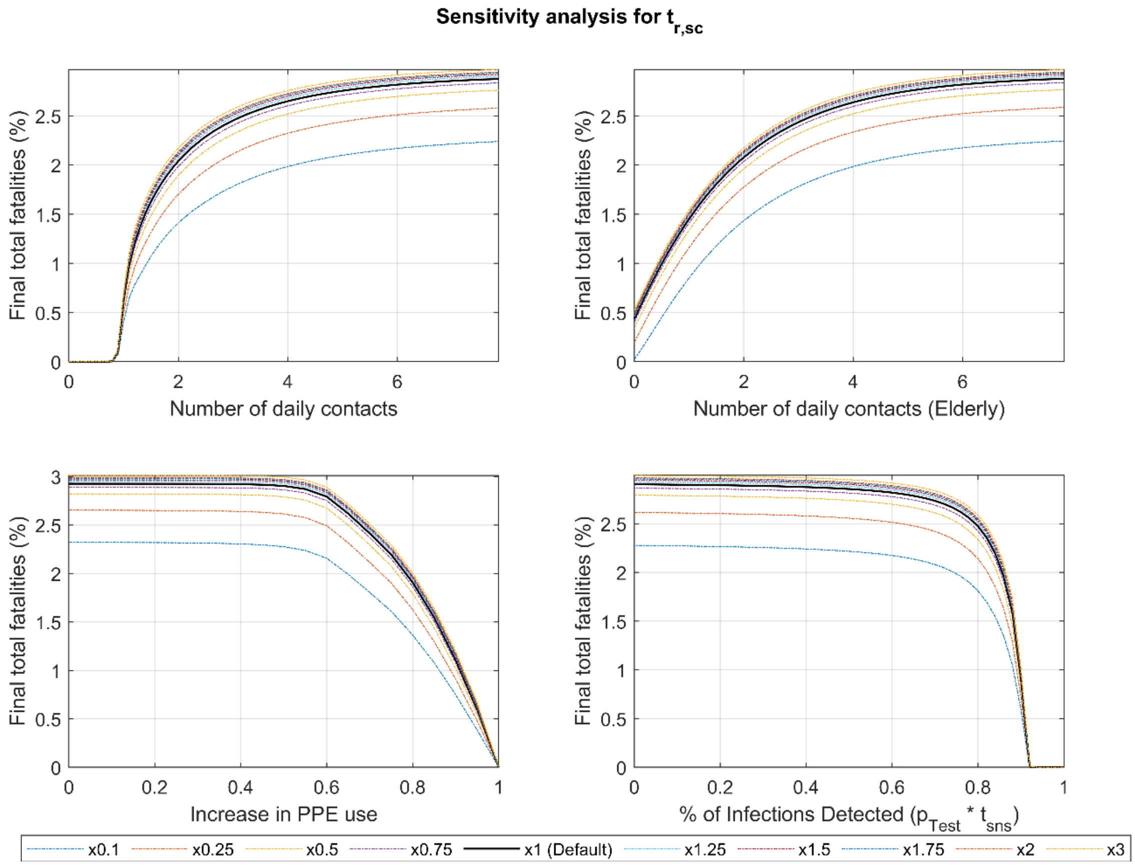

Figure S10.13. Sensitivity analysis for $t_{r,sc}$. The value used for the parameter is the one shown in Table S1 multiplied by the corresponding value shown in the legend.
