# Supplementary material for: Modelling the impact of interventions on the progress of the COVID-19 outbreak including age segregation
Source: PLoS One. 2021 Mar 15;16(3):e0248243. doi: 10.1371/journal.pone.0248243 (PMC7959345; doi:10.1371/journal.pone.0248243)
Supplement: Attachment — Submitted filename: Review of Modelling the Impact of Interventions on the Progress of the COVID.pdf [file pone.0248243.s003.pdf]

Review of Modelling the Impact of Interventions on the Progress of the COVID-19 Outbreak Including Age Segregation

Dear Authors,

“Modelling the impact of interventions on the progress of the COVID-19 outbreak including age segregation” is a very extensive manuscript which includes a mathematical model that incorporates age distribution into the spread of COVID-19 using epidemic data from Spain.

After reviewing the concerns from previous reviewers and the defense from the authors, I’m pleased to see a refined version of the manuscript. I am also convinced by the responses by the authors to the previous reviewer’s comments, especially the comments on the viability of the mode itself (4 and 5 from Reviewer #1).

I have a few comments and suggestions for the authors about the writing:

1. Please conduct additional editing and grammar checking. There are a few instances that I caught that need some slight changes:
    a. Line 409 – “save up significant lives”
    b. Line 49 -- “presented and aimed”
    c. Lines 289-291
2. The discussion on the “dynamic reproduction number”. I’ve never heard this term used before. I think the authors mean the effective reproduction number.
3. Line 69: I would remove the sentence concerning the incorrect reporting of the reproduction number and instead include the actual definition of both the effective reproduction number and the basic reproduction number.
4. Line 400: Include what country the data is coming from again.
5. Include the references for the parameter values in the article – line 284.
6. (lines 327-338) Figure 4, as discussed in the paper implies that selectively choosing to isolate the elderly reduces the final total of fatalities. I didn’t see any explanation of why this happens in the article and I think it should be discussed. A few explanations and questions come to mind:
    a. Perhaps there’s a larger proportion of elderly for that data set? In which case some results may not generalize like the paper is saying.
    b. Has this phenomenon been seen in other data sets?
    c. Does the model assume elderly are more prone to die if they become infected?
