# Supplementary material for: Modelling the impact of interventions on the progress of the COVID-19 outbreak including age segregation
Source: PLoS One. 2021 Mar 15;16(3):e0248243. doi: 10.1371/journal.pone.0248243 (PMC7959345; doi:10.1371/journal.pone.0248243)
Supplement: Attachment — Submitted filename: Response-to-reviewersR2.pdf [file pone.0248243.s004.pdf]

# Response to the Reviewers of the Manuscript
# "Modelling the impact of interventions on the progress of the COVID-19 outbreak including age segregation"

Dear Editor,

We appreciate the opportunity to finalize the manuscript with the reviewer's final important minor comments.

Please find our responses with the details of the corrections below:

**Reviewer #3**:

Dear Authors,

"Modelling the impact of interventions on the progress of the COVID-19 outbreak including age segregation" is a very extensive manuscript which includes a mathematical model that incorporates age distribution into the spread of COVID-19 using epidemic data from Spain.

After reviewing the concerns from previous reviewers and the defense from the authors, I'm pleased to see a refined version of the manuscript. I am also convinced by the responses by the authors to the previous reviewer's comments, especially the comments on the viability of the mode itself (4 and 5 from Reviewer #1).

I have a few comments and suggestions for the authors about the writing:

1. Please conduct additional editing and grammar checking. There are a few instances that I caught that need some slight changes:

a. Line 409 – "save up significant lives"

b. Line 49 -- "presented and aimed"

c. Lines 289-291

These grammatical errors have been corrected.

2. The discussion on the "dynamic reproduction number". I've never heard this term used before. I think the authors mean the effective reproduction number.

3. Line 69: I would remove the sentence concerning the incorrect reporting of the reproduction number and instead include the actual definition of both the effective reproduction number and the basic reproduction number.

This terminology inaccurate use has been corrected we agree now that "effective reproduction number "is the correct term. A line with the definitions of both terms has now been added to address fully this.

4. Line 400: Include what country the data is coming from again.

This has been added.

5. Include the references for the parameter values in the article – line 284.

This has been included now.

6. (lines 327-338) Figure 4, as discussed in the paper implies that selectively choosing to isolate the elderly reduces the final total of fatalities. I didn't see any explanation of why this happens in the article and I think it should be discussed. A few explanations and questions come to mind: a. Perhaps there's a larger proportion of elderly for that data set? In which case some results may not generalize like the paper is saying.

b. Has this phenomenon been seen in other data sets?

c. Does the model assume elderly are more prone to die if they become infected?

Indeed as it is widely known the elderly exhibit a much larger mortality per infection, the model captures this from the beginning since the mortality and severity progression parameters are age specific based on the published data from Spain and others as referenced in the Table S1. This age specific differences captured this way are one of the strengths, we believe, of this work.

Line 335 in the marked-up manuscript should clarify this explanation.

The high mortality of the elderly per infection is widely known to be a universal property of the covid19 disease. The much larger sample size of cases and statistics worldwide show these differences to be clear.

We thank again the reviewer for the useful comments that have helped us make the manuscript of higher quality and clarity.
