# Supplementary material for: Modelling the impact of interventions on the progress of the COVID-19 outbreak including age segregation
Source: PLoS One. 2021 Mar 15;16(3):e0248243. doi: 10.1371/journal.pone.0248243 (PMC7959345; doi:10.1371/journal.pone.0248243)
Supplement: Attachment — Submitted filename: Response-to-reviewersR1.docx [file pone.0248243.s002.docx]

**Response to the Reviewers of the Manuscript**

**“Modelling the impact of interventions on the progress of the**

**COVID-19 outbreak including age segregation”**

Dear Editor,

We are grateful for the opportunity to amend the manuscript and to the reviewers for the time spent in their assessment and for the comments/improvements suggested. We have addressed carefully all the reviewers’ comments and we found their suggestions valuable and resulted in a substantially improved manuscript in our opinion.

As suggested by both reviewers, the abstract and introduction required of major rewriting and we have done so. In addition, please find below the answers provided to the comments by each reviewer.

**Reviewer #1**: The authors addressed an interesting subject of global health importance. COVID-19 interventions were evaluated through a mechanistic model. The authors compartmentalized age groups to assess impact of COVID-19 interventions on the dynamics of spread of the disease, and the final size of the epidemic.

I commend the authors for their efforts. However, I have some major concerns:

1. The Abstract is too long and unfocused; same goes for the Introduction section. Most of the information in the introduction are too generic with no direct relevance to COVID-19.
   The study is assesses the impact of different interventions on COVID-19 spread, so, one would expect the introduction section to include a brief discussion of the literature on modelling studies that have done similar investigation.

We agree that we have fallen short in terms of adequate organisation of the ideas and that reflected particularly on the abstract and introduction, which were in need of a major change. This has been tackled in this revised version we think now it is focused more clearly on the problem at hand, also providing references to more recent publications that have tackled the COVID-19 modelling issue recently.

2. The model development is confusing and the notations are not intuitive - too many subscripts in some of the state variables and parameters. For instance, notations such as rsh_s will be difficult to follow.

We are aware of this and somehow struggled to find the best balance between clarity and rigour in the names of the variables. We have added a line in the materials and methods section to define that the notation r_i,j_ correspond to the rate of the individual going from compartment *j* to compartment *i* to clarify this.

3. Some of the model assumptions do not make practical/biological sense. For example, the model assumes that all critical cases without access to ICU will result in fatality. This is not justifiable, and it will obviously overestimate the fatality rate. Additionally, presymptomatic cases, by definition, will present with symptoms at some point in the future. The model assumes that presymptomatic individuals can recover without intervention.

In regard to the critical cases, we assume it as justifiable. Other works (Giordano et al., 2020) have tackled this issue by increasing the death rate in case that the number of critical cases are over the healthcare capacity. In our case, we assumed that when ICUs are overloaded, a medical decision is taken to leave without ICU care those with lower chances of ICU survival in favor of those with higher chances of ICU survival based on the average ratios of each range. Under this perspective the model assumes that all individuals requiring critical care and not getting it will result in a fatality. The argument could be more refined if accurate data were available but it is only relevant when the ICU capacity is exceeded on the simulations and is not expected to change the conclusions and results significantly. In any case if fatalities without ICU would be lower than 100% there will be still a very high number and the quantitative impact on the results and conclusions will likely change little.

On the second point, we agree with the reviewer that, by defining them as pre-symptomatic is misleading. Therefore, we have renamed them as asymptomatic non-infectious and asymptomatic infectious. Then, an asymptomatic infectious individual can either recover or develop symptoms. This in our opinion describes well the observations of the evolution of the disease in some individuals. This suggested change although only semantic and with no impact in the model equations and results, it is well pointed, and we have changed it accordingly.

4. The model and result discussion are highly speculative. Very few references were made to previous works on COVID-19 modelling in the Discussion. Moreover, the results do not support most of the claims in the discussion section.

We have added now several references (also stated in the revised introduction) that support the claims we added in the discussion section.

For the most speculative parameters believed to be uncertain we conducted sensitivity analyses that are included in the Supplementary material to provide supporting information to that uncertainty. Parameters considered of highest uncertainty include e.g. the level of self-protection and awareness (related to use of PPE), for which assumptions are made.

Although the quantitative the results involved uncertainty as we are dealing with a complex system that adapts to the evolution of the disease, the results and comparisons between types of interventions respect to each other is the key message. The limitations of the model have been clearly outlined to be kept them in mind in the discussion.

We agree the manuscript feel behind in referencing previous works on COVID-19 modelling, this was partly due to the fast pace of publications in the last months and manuscript revision delays after submission to journals. We have amended this issue to reflect as up to date as possible.

Although our results support the conclusions we agree as it has been pointed out by Reviewer #2 as well, that we fell short in clarity. This has been amended. We have double checked the model against any flaws or code bugs and found nothing contradicting the assumptions and structure as it is described in the paper. We explain specific comments below:

5. The flaws in the model development and assumptions became more apparent in the results: in figure 4 for example, it is strange that the % of fatality is higher than % of critical cases. This is clearly wrong, since fatality is a subset of critical cases. Moreover, it is also suspicious that you have 0 critical cases if the number of contact per day is about 0.9. Additionally, 50% increase in PPE has no effect on the final total fatality? 90% of case detection will lead to 0 fatality? These can’t be right.

After careful revision we are convinced that the model structure and assumptions are well reflected in the results and no such flaws have been found. What we realise however based on the comments is that we may have failed to explain what was plotted or represented in some of the results and could have been misinterpreted due to our shortcoming. Below specific explanations for the comments raised for each figure:

- Figure 4: We consider that the results are correct. What we are representing as the caption of figure 4 states is the total cumulative fatalities on the left plot and on the right side, the **maximum number of critical cases** that ever occurred during one simulation. Therefore, it is expected that the total fatalities is higher than the single point in time of critical cases. This becomes clear in the supplementary material where all plots of fatalities and number of critical cases over time for all interventions are shown, so they can be also compared. In this case, please see the simulation over time for the universal social isolation case.

**Figure S6.a**. Impact of universal social isolation on the final total number of fatalities (top left); the final total number of fatalities per age group (top right) as well as for the different time course profiles of the total active cases (middle left); reproduction number (R_t_) (middle right); the number of critical cases (bottom left) and the number of fatalities (bottom right). Numbers are as percentage of total population of all ages.

The number of critical cases suggested as zero is actually just very low, around 10^-4^ %. In populations of millions will result in few critical cases. This is understandable considering that for that level of protection of the people and with the people basically isolated, the spread of the disease is practically collapsed and there is basically no transmission of the virus. Our starting point of degree of social interaction was 10, and 0.9 represents more than a 90% decrease of interaction for all groups between themselves. Given that it is a multiplicative process, it is expected that at some values the spread of the disease collapses.

- In figure 5, we estimate PPE use has no impact on the final total fatalities indeed. The outbreak seems to slow down (Figure S7 in the supplementary material), but eventually the same number of fatalities is reached at values of 50% or lower. At the end, the result is dependent on how long the ICU capacity is exceeded. Levels of increase in lpa below 50% show lower peaks of critical cases. However, the area below the curve is almost similar and therefore eventually resulting in similar deaths.
- Figure 6: It appears that when testing and identification of all individuals is of 90% success the curves are slowed down so much that you can quarantine all pre-symptomatic. This can be again observed in Figure S8c, in Appendix VIII in the supplementary material. Again, as in figure 4, this is a multiplicative process, and it means that when some values increase or decrease their impact is magnified. However, the capacity to detect 90% of the infections is nearly impossible, and we are moving in a range around de 80% at best. We have added this explanation in the discussion for better clarification.

We have added in the discussion of all the interventions proper justifications of the observations linking to the supplementary material, in order to provide much more clarification if it is needed. But looking at both results on Figures 4-6 and in the Supplementary Material, the results appear to be fully correct to us in relation to the model assumptions.

**Reviewer #2**: The authors have modeled the spread of a Covid-like infection across a population, the population being divided into 9 age groups from infancy to old age and the disease stage depicted from asymptomatic and pre-symptomatic to deceased. Equations are set up for transitions across disease stage in time. Real data was used to evaluate the parameters in the model. It is not clear that interventions such as isolation etc. were modeled as stated. It is claimed that the model is deterministic, but it is possible that there could be chaotic outcomes especially with multiple interacting equations.

In our opinion the model is fully deterministic, the nature of the differential equations quasi linear almost ensures no chaotic effects are expected. The model contains no random variables or parameters that cannot in principle be measured or estimated to a single average representative number as per the model definitions and assumptions as described. It is however of no specific interest to us to dwell on the semantics of the model classification neither have we done a thorough mathematical analysis of its equations and mathematic characteristics, therefore we have removed unnecessary references to the deterministic or not nature of the model from manuscript.

We have reorganised the materials and methods section in an attempt to clarify better how the interventions were modelled. We have revised the consistency between what is explained and what was implemented and the source code is made available. In addition, extensive plots have been added in the supplementary material to enhance clarity and transparency.

To be reconsidered for publication

(1) the content can be made more crisp and clear.

Major sections of the manuscript have undergone major rewriting as we agree with the reviewers that it was necessary.

(2) the length reduced by about half

As suggested, we agree and have decreased significantly the length of the abstract, the introduction and the discussion sections for this purpose. Now the paper stands as 80% of the original length. Although our current choice was to keep the model description in the main body, it is possible to move it into the supplementary information section if required by the editor.

(3) bring out the conclusions and interpretation more clearly

In line with the above we agree that this and other sections fell short in clarity and this has been addressed.

(4) summarize the conclusions making reference to the claims set forward in the abstract

The redoing of the abstract and is now better aligned with the conclusions as well pointed by the reviewers.
